# Using Nightly Sleep Guidelines to Address Links Between Adolescents’ Self-Reported Weekly Sleep Patterns and Anxiety and Depression Symptoms

**DOI:** 10.1007/s10578-023-01610-0

**Published:** 2023-10-12

**Authors:** Allison M. Waters, Lily Gibson, Rachel A. Sluis, Kathryn L. Modecki

**Affiliations:** School of Applied Psychology and Centre for Mental Health, Griffith University, Mount Gravatt Campus, Brisbane, Australia

**Keywords:** Sleep duration, Weekly sleep patterns, Anxiety and depression

## Abstract

Short and long nightly sleep durations are associated with anxiety and depression symptoms among adolescents. However, studies have not used recommended hours of nightly sleep or focused on sleep patterns across weekdays and weekends in examining links with anxiety and depression symptoms. The present study included 709 adolescents in Grade 11 (402 females; 307 males) who self-reported hours of sleep on weeknights and weekends and anxiety and depression symptoms. Using the recommended 8–10 h to define average nightly sleep for adolescents, sleep patterns across weekdays and weekends were categorised into seven classes: short stable, short increasing, average decreasing, average stable, average increasing, long decreasing, and long stable. Relative to average stable sleepers, short stable, short increasing, and long stable sleepers had significantly higher anxiety and depression. Adolescents require 8–10 h of sleep on weeknights, regardless of weekends, for optimal emotional wellbeing.

## Introduction

Anxiety disorders and major depression disorder are the most common mental health disorders among adolescents, with 4.6% of 15–19 year-olds experiencing an anxiety disorder and 2.8% of 15–19-year-olds suffering from major depression in the US, Australia, and elsewhere in the world [1–3]. Not only does anxiety and depression in adolescence affect social, educational, and personal functioning, but they also predate and predict mental health problems in adulthood [4] and are associated with continued long-term social, occupational, and relationship problems [5]. As a result, research on modifiable factors that are associated with elevated internalising problems during the adolescent period is especially important for informing practice and policy.

Notably, government health departments and national sleep organisations provide expert guidance for policy makers and practitioners, based on formative research on optimal sleep duration for adolescents [6]. For example, the Australian Government and the American Academy of Sleep Medicine recommend that adolescents between 14 and 17 years of age attain between 8 and 10 h of sleep each night and should sleep no fewer than 7 h or more than 11 h per night [7, 8]. Despite the aim of guiding recommendations for appropriate sleep, government and/or national sleep organisation recommendations have rarely been used in studies to define short, average, and long nightly sleep durations and subsequent associations with mental health. Instead, researchers have generally identified nightly sleep durations overall, which are associated with varying levels of emotional symptoms, either in large samples of school-age children or by comparing self-reported nightly sleep durations in clinically anxious or depressed adolescents relative to healthy controls. However, from a health promotion perspective, these approaches limit our ability to identify and communicate *specific* nightly sleep durations associated with optimal versus impaired emotional wellbeing among adolescents.

Overall, past research has found that reduced and excessive nightly sleep durations are generally associated with anxiety, depression, and co-morbid anxiety and depression among adolescents (e.g., [9, 10]). For example, several studies examined hours of sleep per night and anxiety symptoms in large samples of high school aged adolescents. They report that higher anxiety symptoms were associated with sleeping 6.5 h or less [11], 7 h or less [12] or 7.5 h or less, regardless of gender [13]. Similarly, research conducted with clinically anxious adolescents aged 11–17 years with anxiety disorders found that short sleep durations (fewer than 6 h per night) increased the severity, frequency, and maintenance of anxiety symptoms [14].

Studies reporting on long nightly sleep duration and anxiety symptoms are fewer in number, and findings are mixed. For example, higher anxiety symptoms have been found among adolescent males who reported sleeping 9.5 h or more relative to males who slept between 8.5 and 9.5 h and by females who reported sleeping 8.5 h or more relative to females who slept between 7.5 and 8.5 h per night [13]. However, the range of 8.5–9.5 h still falls within the recommended average range of 8–10 h of sleep per night recommended by government and sleep medicine organisations. Thus, it is not clear whether these differences represent meaningful variation in average sleep per night, nor how these adolescents would differ if classified according to best practice guidelines. In terms of long nightly sleep, no studies have reported on clinically anxious compared with healthy adolescents. However, in research with clinically anxious adults, sleeping more than 10 h has been associated with elevated anxiety symptoms [15].

Similar to anxiety, reduced sleep duration is commonly observed in adolescents with elevated depression symptoms. However, similar lack of congruence regarding definition differences in nightly sleep duration apply. Numerous studies have examined large samples of adolescents between 11 and 19 years of age and found that adolescent self-reported sleep of 6 or fewer hours per night and 7 or fewer hours per night also report elevated depression symptoms relative to sleeping more than 8 h per night (e.g., [16–18]). Similarly, Roberts et al. [19] examined clinically depressed adolescents aged 11–17 years and found that short sleep duration fewer than 6 h per night increased depression symptoms over a 1-year period. In longitudinal studies, nightly sleep duration or fewer than 8 h per night differentiated depressed from anxious and healthy adolescents and at 15 years of age significantly predicted the severity of both anxiety and depression symptoms at age 21 (e.g., [20]).

Surprisingly few studies have examined longer sleep durations and depression symptoms among adolescents specifically. Several studies have found short duration sleep of fewer than 7–8 h per night *and* long sleep duration of more than 8–9 h per night were associated with depression symptoms in samples spanning children through older adults (e.g., [21–23]). However, the delineation between short and long sleep durations has cut across national recommendations of 8–10 h of sleep for adolescents making it difficult to determine the salience of recommended guidelines for depression among adolescents.

In fact, very few studies have applied pre-determined sleep duration intervals to examine the association of sleep and mental health status among adolescents, regardless of whether they align with government and/or sleep medicine organisation recommendations. With Japanese adolescents aged 12–18 years of age, Kaneita et al. [24] used interval cut-offs of fewer than 5 h, 5–6 h, 6–7 h, 7–8 h, 8–9 h and more than 9 h per night and observed a U-shape association. Here, sleeping fewer than 7 h per night or more than 9 h per night was associated with higher mean scores of general mental health compared to adolescents sleeping between 7 and 9 h. However, anxiety and depression were not examined specifically. In a more recent study, Ogawa et al. [25] also used predefined sleep intervals and found that those adolescents “without adequate sleep duration” of less than 8 h per night reported higher anxiety and depression symptoms compared to those attaining an “adequate sleep duration” of between 8 and 10 h per weeknight. However, this study excluded adolescents who slept more than 10 h per night.

Notably, a critical limitation of past research has been that most studies have failed to consider differing sleep patterns across the course of the week. This is an important consideration, given that adolescents have been found to maintain different sleep patterns during weekdays and weekends with increasing age relative to other age groups [12, 26, 27]. This observed shift is likely due to biological influences, as well as extracurricular, social and educational obligations which require adolescents to wake up early and stay up late, and thus, contribute to varied sleep patterns across the week [28, 29]. Indeed, some adolescents may accumulate a ‘sleep debt’ and compensate for this with longer sleep durations on the weekend [30, 31].

However, to date, the relationship between changes in adolescents’ nightly sleep duration during the week and over the weekend and associations with mental health symptoms remains highly unclear. Among those few studies considering sleep patterns across the week, results are mixed. Some studies have found that both short and long periods of sleep over the weekend were associated with increased odds of mood disorders (e.g., [32]). Through a systematic review of 6- to 24-year-olds, Sun et al. [33] also found that sleep duration differences ranging from more than 2–4 h on the weekend relative to weekdays was positively correlated with depression symptoms compared to adolescents who slept consistent durations across the week. On the other hand, among 12–18-year-olds, Lee et al. [34] found that “catch up” sleeping on the weekend may be more beneficial for adolescent’s depression symptoms than having consistently short sleep, between 5 and 7 h across the week. Thus, further research is required to clarify how full weekly sleep patterns relate to adolescents’ emotional health.

As alternative approach has been considered from the perspective of maintaining stable sleep across the week. However, similarly few studies have examined the effects of different stable patterns of short, average, and long nightly sleep durations and they have rarely made comparisons with variable patterns of nightly sleep durations across the week. Hirshkowitz et al. [6] examined the benefits of stability in weekly sleep durations on mental health for adolescents aged 14–17 and concluded the most beneficial sleep durations for the health and wellbeing of adolescents was stable sleep across the week of 8–10 h per night. However, given that adolescence is a time of increasing social, recreational and extra-curricular engagements that are likely to impact sleep stability, further studies are required to determine whether variation in sleep duration on the weekend after a week of stable sleep of 8–10 h per night has differential effects on anxiety and depression symptoms.

In summary, evidence suggests that short sleep durations and, possibly, long sleep durations are associated with increased anxiety symptoms (e.g., [11, 14]) and that both short and long sleep durations are associated with depression symptoms in adolescents [16]. However, at the same time, the hours of sleep identified cut across the hours of sleep recommended for adolescents in available guidelines, such as those proposed by the Australian Government [8]. This makes it difficult to pinpoint how recommendations play out in relation to adolescent mental health. In particular, whether different patterns of sleep across the week are associated with increased anxiety and depression symptoms still remains unclear. As a result, the current study applies the Australian Government recommended sleep durations for adolescents to address whether different possible weekly sleep patterns are associated with anxiety and depression symptoms among adolescents.

In the current study, following the Australian Government guidelines, seven different sleep classifications across the week were created. These included short stable sleep (less than 8 h on weekdays and weekends) and short increasing sleep (less than 8 h on weeknights and more than 8 h on weekends); average decreasing sleep (8–10 h on weeknights and less than 8 h on weekends), average stable sleep (8–10 h on weeknights and weekends), and average increasing sleep (8–10 h on weeknights and more than 10 h on weekends); as well as long decreasing sleep (more than 10 h on weeknights and less than 10 h on weekends) and long stable sleep (more than 10 h on weeknights and weekends).

Based on the Australian Government sleep duration recommendations [8], the ‘reference group’ in the present study was average stable sleepers of 8–10 h per night across the week. Here, it was hypothesised that the other weekly sleep patterns would significantly vary in their association with anxiety and depression. Specifically, because catch-up sleeping on the weekend after a week of short sleep durations [31] and stable short sleep durations throughout the week have both been associated with high levels of anxiety symptoms [11–13] and depression symptoms [16, 18], it was hypothesised that short stable and short increasing weekly sleep patterns would be associated with elevated anxiety and depression symptoms compared to average stable sleepers. Additionally, among adolescents who sleep between the average recommended sleep durations of 8–10 h on weeknights (short stable sleep, short increasing sleep), it was hypothesised that increases, decreases, or stable sleep over the weekend would not be associated with differential anxiety or depression symptoms [6]. Furthermore, it was hypothesised that the long stable sleep patterns throughout the week and decreasing on the weekend (more than 10 h on weeknights and less than 10 h on weekends) would be associated with elevated anxiety and depression symptoms relative to average stable sleepers, given that long sleep durations have generally been associated with increased anxiety and depression symptoms [16, 32]. Finally, based on previous studies of short sleep duration [14, 16] and long sleep duration [13], the final hypothesis was that short stable and long stable sleep patterns would be associated with higher anxiety and depression symptoms relative to average stable sleepers.

## Methods

### Participants

Participants in the present study were 815 Grade 11 high school students attending two single-sex schools in Australia. The school-level socio-economic status for the schools (Index of Community Socio-Educational Advantage; ICSEA) for the schools were slightly above the average educational advantage of these student populations (ICSEA’s range from 500 to 1300, with 1000 the median and a SD of 100, both schools were in the range of 1100). Adolescents with parental consent completed the study during school hours.

Adolescents participated in the study between 2019 and 2021; 411 females (*M*_*age*_ = 16.13, *SD* = 0.45), 314 males (*M*_*age*_ = 16.55, *SD* = 0.53), 21 other (*M*_*age*_ = 16.38, *SD* = 1.47) and 69 unspecified. Due to missed data collection on the day of assessment, the final sample size for the current study was 709 participants, 402 females (*M*_*age*_ = 16.13, *SD* = 0.45) and 307 males (*M*_*age*_ = 16.55, *SD* = 0.53). Table 2 presents descriptive information about the sample and indicates that most participants were born in Australia, spoke English and lived at home with both parents.

### Measures

#### Weeknight and Weekend Night Sleep Duration

Students completed two items relating to sleep duration on weeknights (e.g., “How many hours of sleep would I usually get on a weekday night?”) and weekend nights (e.g., “How many hours of sleep would I usually get on a weekend night?”). Weeknights and weekend nights were not predefined in the question in order to permit adolescents to respond based on their own evaluation of which nights represent weekdays and weekends and in accordance with past research [35, 36]. Responses were rated on an 8-point scale of 6 = 6 h or less, 7 = 7 h, 8 = 8 h, 9 = 9 h, 10 = 10 h, 11 = 11 h, 12 = 12 h, 13 = 13 h or more.

#### Weekly Sleep Pattern

Seven different weekly sleep pattern classifications were then computed based on responses to the two items assessing weeknight and weekend sleep duration. Sleep pattern groups were defined based on the Australian Government recommendation of 8–10 h of sleep per night for 14–17 year olds [8], with additional reference to previous research on short sleepers (e.g., [11]), catch-up sleepers (e.g., [31]) and long sleepers (e.g., [37]). Therefore, Table 1 presents the sleep pattern classifications that were established based on adolescents’ responses to the two items relating to weekday and weekend nightly sleep durations.

**Table 1:** Description of weekly sleep pattern categories

Weekly sleep pattern	Definition
Short stable	Fewer than 8 h of sleep across weekdays and the weekend
Short increasing	Fewer than 8 h of sleep during weekdays and more than 8 h of sleep on the weekend (i.e., catch up sleeping)
Average decreasing	8–10 h of sleep during weekdays and fewer than 8 h of sleep on the weekend
Average stable	8–10 h of sleep across weekdays and the weekend
Average increasing	8–10 h of sleep during weekdays and more than 10 h of sleep on the weekend
Long decreasing	More than 10 h of sleep during weekdays and fewer than 10 h of sleep on the weekend
Long stable	More than 10 h of sleep across weekdays and the weekend

#### Anxiety and Depression Symptoms

The Revised Children’s Anxiety and Depression Scale—Short Version (RCADS; [38]) was used to assess adolescents’ anxiety and depression symptoms. The RCADS consists of 25 self-report items and produces total anxiety and depression scores. Responses are provided using a four-point Likert scale (0 = never, 1 = sometimes, 2 = often, 3 = always) to assess anxiety symptoms (15 items) (e.g., “I worry when I think I have done poorly at something”) and depression symptoms (10 items) (e.g., “I feel sad or empty”). Higher scores on each subscale indicated higher levels of anxiety and depression. Cronbach’s α were 0.86 for the anxiety subscale and 0.90 for the depression subscale.

### Procedure

Ethical approval was granted through University Human Research Ethics Committee as part of a larger study on youth mental health and wellbeing (Ref: 2017/858). Letters were sent to principals of participating schools and following approval, school personnel sent letters to parents providing information about the study and to obtain parent consent. For students with parent consent, the link to an online survey was distributed to the participating adolescents via email and completed during one session during school hours. The sleep items and RCADS in the present study were completed as part of a larger survey. After completion of these measures, information was sent automatically to the research team for data screening and analysis. A report on student mental health and wellbeing of students was provided to school personnel who in turn provided feedback to parents and students.^1^

### Data Screening and Analyses

#### Data Screening

Of the 815 participants recruited, 69 were absent on the day of the assessment and a further 17 participants did not complete the assessment due to extra-curricular activities at the time of the assessment. Because of the very small sample size and difficulties classifying sleep and mental health outcomes according to past research, data were omitted for the 20 participants who selected “other” as their sex. The final sample consisted of N = 709 participants which included 402 females and 307 males.

#### Data Analysis

Chi-square tests were conducted to examine categorical demographic variables including country of birth (Australia vs other), language spoken at home (English vs other), and living arrangements (both parents v other) between each weekly sleep pattern group and the reference group being the average stable sleepers. One-way ANOVAs and chi-square analyses were performed to examine age and sex differences among weekly sleep pattern groups in relation to anxiety and depression symptoms. To analyse the association of weekly sleep patterns with total anxiety and depression symptoms scores, analyses of covariance (ANCOVA) were performed using sex as a covariate given sex differences in anxiety and depression symptoms and prior observed differences in sleep duration as a function of sex among adolescents [39] and sleep pattern group as the independent variable. Partial eta-squared is reported as a measure of effect size. Follow-up comparisons were conducted using Tukey–Kramer post-hoc tests.

## Results

### Initial Control Analyses

Table 2 displays the descriptive information for all sleep pattern groups. Chi-square analyses confirmed there were no significant differences in the number of males and females in each sleep pattern group compared to the reference group of average stable sleepers, all *p* values > 0.05. Chi-square tests were also performed to compare country of birth (Australia vs other), language spoken at home (English vs other), and living conditions (both parents vs other), for each sleep pattern group relative to average stable sleepers and revealed no significant differences, all *p* values > 0.05.

**Table 2:** Descriptive information as a function of sleep pattern categories

	Short stable (n = 149)	Short increasing (n = 229)	Average decreasing (n = 80)	Average stable (n = 152)	Average increasing (n = 66)	Long decreasing (n = 10)	Long stable (n = 23)
Age (mean (*SD*))^a^	16.42 (0.55)	16.28 (0.48)	16.34 (0.48)	16.28 (0.48)	16.27 (0.45)	16.00 (1.16)	16.30 (1.06)
Sex							
% Female	49.7%	63.8%	50.0%	61.2%	54.5%	70.0%	56.1%
Country of birth							
% Australia	87.9%	88.2%	88.8%	89.5%	83.3%	70.0%	87.0%
Language spoken at home							
% English	96.0%	95.6%	97.5%	98%	98.5%	90.0%	82.6%
Living conditions							
% Living at home	85.9%	90%	85.0%	89.5%	83.3%	80.0%	65.2%
Living with							
% Both parents	81.9%	78.6%	80.0%	78.3%	66.7%	80.0%	69.7%
Weeknight sleep duration—mean (*SD*)^b^	6.60 (0.49)	6.76 (0.44)	8.21 (0.41)	8.24 (0.43)	8.30 (0.46)	10.40 (0.97)	11.35 (1.37)
Weekend night sleep duration—mean (*SD*)^b^	6.47 (0.50)	9.00 (1.15)	6.76 (0.43)	8.53 (0.50)	10.21 (0.62)	8.20 (1.03)	11.87 (1.18)
RCADS anxiety total—mean (*SD*)^b^	12.43 (7.88)	12.01 (6.55)	8.81 (5.80)	9.66 (5.10)	8.88 (6.05)	7.90 (6.23)	14.43 (12.77)
RCADS depression total—mean (*SD*)^b^	12.21 (7.06)	11.63 (5.84)	8.46 (5.33)	7.90 (5.05)	7.89 (4.67)	7.10 (4.70)	10.09 (9.01)

^a^Years: months

^b^Hours: minutes

A one-way between-subjects ANOVA conducted to examine differences in age between weekly sleep pattern groups was not significant, *F*(6, 708) = 2.09, *p* = 0.053, *n*^2^ = 0.02. However, one-way between-subjects ANOVAs conducted to examine gender differences in anxiety and depression symptoms were significant, *F*(1, 708) = 19.27, *p* < 0.001, *n*^2^ = 0.03 and *F*(1, 708) = 40.09, *p* < 0.001, *n*^2^ = 0.05, respectively with females reporting significantly higher anxiety and depression than males.

### Anxiety Symptoms

Table 2 displays the average anxiety symptoms for each weekly sleep pattern group. The 7 (sleep group: short increasing, short stable, average decreasing, average stable, average increasing, long decreasing, long stable) × 2 (sex as a covariate: female or male) ANCOVA, revealed a significant main effect for sleep group, *F*(6, 709) = 3.61, *p* = 0.002, *η*_*p*_^2^ = 0.03, and a non-significant main effect for the covariate of gender, *F*(1, 709) = 2.03, *p* = 0.155, *η*_*p*_^2^ = 0.003. The interaction between sleep group and sex was not significant, *F*(6, 709) = 0.90, *p* = 0.495, *η*_*p*_^2^ = 0.01.

#### Weekly Short Sleep Patterns and Anxiety Symptoms

Post-hoc comparisons using the Tukey–Kramer test confirmed that short stable and short increasing sleepers had significantly higher levels of anxiety compared to the average stable sleepers, *p* = 0.009 and *p* = 0.02 respectively. Additionally, short stable and short increasing sleepers did not significantly differ in levels of anxiety symptoms, *p* = 0.997 (see Table 2). Therefore, regardless of any changes in sleep duration on the weekend, adolescents who had short sleep durations of fewer than 8 h per night during the week were more likely to have higher levels of anxiety compared to adolescents who slept the recommended 8–10 h of sleep per night throughout the week.

#### Weekly Average Sleep Patterns and Anxiety Symptoms

Post-hoc comparisons using the Tukey–Kramer test confirmed that there were no significant differences in anxiety symptoms between average stable sleepers, average decreasing sleepers (*p* = 0.974) and average increasing sleepers on the weekend (*p* = 0.988).

#### Weekly Long Sleep Patterns and Anxiety Symptoms

Post-hoc comparisons using the Tukey–Kramer test confirmed that long stable sleepers had significantly higher levels of anxiety compared to the average stable sleepers, *p* = 0.033, and long decreasing sleepers, *p* = 0.011. However, contrary to hypotheses, there were no significant differences in anxiety levels between average stable sleepers and long decreasing sleepers, *p* = 0.985. Therefore, adolescents who had long sleep durations of more than 10 h per night throughout the week were more likely to have higher levels of anxiety compared to adolescents who slept the recommended 8–10 h of sleep per night throughout the week or on the weekends.

#### Weekly Stable Sleep Patterns and Anxiety Symptoms

Post-hoc comparisons using the Tukey–Kramer test confirmed that short stable and long stable sleepers had significantly higher levels of anxiety compared to the average stable sleepers, *p* < 0.009 and *p* = 0.033, respectively. Additionally, short stable and long stable sleepers did not significantly differ in levels of anxiety symptoms, *p* = 0.853. Therefore, adolescents who slept for short durations of fewer than 8 h per night, or long durations of more than 10 h per night, across the week were more likely to have higher levels of anxiety compared to adolescents who slept the recommended 8–10 h of sleep per night throughout the week.

### Depression

#### Weekly Sleep Patterns and Depression Symptoms

Table 2 displays the average depression symptoms for each weekly sleep pattern group. The 7 (sleep group: short increasing, short stable, average decreasing, average stable, average increasing, long decreasing, long stable) × 2 (sex as a covariate: female or male) ANCOVA revealed significant main effects for sleep group, *F*(6, 709) = 4.21, *p* < 0.001, *η*_*p*_^2^ = 0.04, and the covariate of sex, *F*(1, 709) = 5.64, *p* = 0.018, *η*_*p*_^2^ = 0.01. However, the interaction between sleep group and sex was not significant, *F*(6, 709) = 1.01, *p* = 0.417, *η*_*p*_^2^ = 0.01.

#### Weekly Short Sleep Patterns and Depression Symptoms

Post-hoc comparisons using the Tukey–Kramer test confirmed that short stable and short increasing sleepers had significantly higher levels of depression compared to the average stable sleepers, both *p* < 0.001. Short stable and short increasing sleepers did not significantly differ in levels of depression symptoms, *p* = 0.966 (see Table 2). Therefore, regardless of changes in sleep duration on the weekend, adolescents who had short sleep durations of fewer than 8 h per night during the week were more likely to have higher levels of depression compared to adolescents who slept the recommended 8–10 h of sleep per night throughout the week.

#### Weekly Average Sleep Patterns and Depression Symptoms

Post-hoc comparisons using the Tukey–Kramer test confirmed that there were no significant differences in depression symptoms between average stable sleepers, average decreasing sleepers (*p* = 0.974) and average increasing sleepers on the weekend (*p* = 0.977). Therefore, any changes in nightly sleep duration on the weekend did not significantly relate to depression symptoms in adolescents who slept the recommended 8–10 h of sleep per night during the week.

#### Weekly Long Sleep Patterns and Depression Symptoms

Post-hoc comparisons using the Tukey–Kramer test confirmed that long stable, but not long decreasing sleepers, had significantly higher depression symptoms compared to average stable sleepers, *p* = 0.048, and *p* = 0.932, respectively. Additionally, long stable sleepers had significantly higher depression symptoms than long decreasing sleepers, *p* = 0.041. Therefore, sleeping more than 10 hours throughout the week was significantly related to higher depression symptoms.

#### Weekly Stable Sleep Patterns and Depression Symptoms

Post-hoc comparisons using the Tukey–Kramer test confirmed that short stable sleepers and long stable sleepers had significantly higher levels of depression compared to average stable sleepers, *p* = 0.001 and *p* = 0.042 respectively. Additionally, long stable and short stable sleep patterns did not significantly differ in levels of depression symptoms, *p* = 0.681. Therefore, adolescents who slept for short durations of fewer than 8 h per night across the week and longer than 10 h per night throughout the week were more likely to have higher levels of depression compared to adolescents who slept between the recommended 8–10 h throughout the week.

## Discussion

The current study examined the relationship between weekly sleep patterns and anxiety and depression symptoms in adolescents using the Australian Government nightly sleep duration recommendations [8] to define different classes of weekly sleep patterns. The results confirmed that short stable sleepers (who slept less than 8 h per night all week), short increasing sleepers (who slept less than 8–10 h during the week and more than 8 h on the weekend), and long stable sleepers (who slept more than 10 h per night throughout the week) had significantly higher levels of anxiety and depression than average stable sleepers (who slept between 8 and 10 h per night throughout the week). Thus, sleeping less than 8 h per night during weeknights, regardless of nightly sleep duration on the weekend, and sleeping more than 10 h every night throughout the week, was associated with the highest levels of both anxiety and depression among adolescents.

The current findings are consistent with, and extend upon, prior studies that have applied varying methods to define and identify short sleep durations among adolescents and reported significant associations between sleep durations varying from 6.5 to 9.5 h per night and elevated anxiety symptoms [11–14] and short sleep durations of fewer than 6–8 h per night and depression symptoms [16, 18, 19]. However, the current study builds on prior studies by considering weekday and weekend sleep patterns and found that regardless of increases or decreases in sleep duration on the weekend, adolescents who reported sleeping fewer than 8 h of sleep per night throughout the weekdays were more likely to have higher levels of anxiety and depression compared to adolescents who slept between the recommended 8 and 10 h per night during the week. Both short and longer periods of sleep on weekends have been found to be associated with mental health disorders in prior studies (e.g., [32]), and findings have been inconsistent in relation to whether changes in sleep duration from weekdays to weekends increases or reduces depression symptoms (e.g., [33, 34]). Moreover, prior studies have not examined weekend nightly sleep durations following shorter relative to longer weeknight durations. Thus, the present findings suggest that, providing adolescents receive 8–10 h of sleep per night during the week, variation in nightly sleep duration on the weekend may not adversely relate to their anxiety and depression levels. Rather, stable short sleep through the weekdays, regardless of changes on the weekend, was found to be associated with increased anxiety and depression symptoms.

In terms of long sleep durations, the present study also extends on limited studies in which long sleep durations of more than 8–9 h per night were associated with elevated anxiety symptoms compared to adolescents who slept fewer than 8–9 h [13]. Moreover, associations of long nightly sleep duration with depression symptoms have been found in some prior studies (e.g., [40, 41]) but not in others (e.g., [20]). However, 8–9 h of sleep per night used in these prior studies to delineate long from short sleep durations falls within the recommended sleep duration of between 8 and 10 h for adolescents proposed by the Australian Government and other sleep medicine organisations [7, 8]. Moreover, prior studies have not examined different combinations of adequate and inadequate sleep on weeknights and weekends and associations with anxiety and depression symptoms. Therefore, the observations in the present study that long stable sleepers attaining more than 10 h of sleep per night across the week was associated with elevated anxiety and depression symptoms highlights the need for further research employing government and/or sleep organisation guidelines before firm conclusions can be made.

Although the present study identified important associations between short and long sleep patterns and anxiety and depression symptoms among adolescents, there are several limitations that warrant consideration. The current study included participants from Australian independent schools that had relatively high SES which limits the generalisability of findings to adolescents across different school districts, cultural contexts, and socio-economic backgrounds. Future studies should aim to apply government and sleep medicine organisation guidelines to examine associations of sleep and anxiety and depression in public school students [42], non-school students [43], and rural students [44]. Another limitation is that due to the way in which participants were allocated to different weekly sleep pattern categories based on their total sleep durations across the week and weekend, there were unequal samples sizes among the sleep pattern groups. Therefore, the study consisted of limited participants in the two long sleep pattern groups in comparison to the other weekly sleep pattern groups. Although this is consistent with prior studies which have reported smaller proportions of long than average and short duration sleepers [13], future studies should aim to recruit more adolescents with long sleep durations to ensure adequately powered analyses. Although the aim of the present study was to examine links between self-reported sleep and anxiety and depression symptoms using the Australian Government guidelines for public health and communication purposes (i.e., 8–10 h per night for 14–17-year-olds, each sleep classification defined in the present study included 2 or more hours of increasing sleep. Future studies should examine whether these incremental increases in sleep duration within classifications are associated with changes in anxiety and depression symptoms. Furthermore, future studies should also assess potential mechanisms linking anxiety and depression symptoms and sleep duration differences (e.g., avoidance behaviour; rumination). The present study was also cross-sectional in nature and future research should use a longitudinal design to examine changes in weekly sleep patterns and anxiety and depression across different ages and developmental stages (e.g., [20, 45]). Additionally, although participant time and burden completing lengthy assessment procedures during class or at home needs to be considered, future studies should consider using self-report and/or parent-report sleep diaries [46] or objective measures such as polysomnography [47], actigraphy or sleep EEGs [48]. Finally, the present study did not specify Monday to Friday as weekdays and Saturday to Sunday as the weekend so that adolescents answered these questions based on their own judgement of their weekend nights. For adolescents, Friday and Saturday may be considered weekend nights rather than Saturday and Sunday [35, 36]. Therefore, a future direction could be to consider differences in adolescent anxiety and depression as a function of weekday sleep defined as Sunday to Thursday and weekend sleep from Friday to Saturday relative to the traditional Monday to Friday and Saturday and Sunday for weekday and weekend classes respectively.

In conclusion, the current study found support for the recommendation that adolescents should attain between 8 and 10 h of sleep per night across the week for optimal levels of anxiety and depression [6]. Shorter and longer nightly sleep durations were detrimentally associated with emotional health, and changes in sleep duration on weekends did not change these associations. The findings provide support for using government and sleep organisation guidelines to provide greater consistency across studies and in public policy and practice to communicate with young people and parents about the importance of adolescents attaining 8–10 h of sleep per night, especially through the week, for good emotional health. Finally, the findings highlight the importance of prevention and early intervention programs (e.g., [49–51]), specifically addressing sleep durations of less and more than 8–10 h per night for improving adolescent emotional health.

## Summary

This study found that relative to adolescents who self-reported sleeping the recommended 8–10 h of sleep per night throughout the week, adolescents with short stable, short increasing, and long stable sleep patterns across weeknights and weekends had significantly higher anxiety and depression. The study provides important support for current adolescent sleep recommendations and supports that adolescents require 8–10 h of sleep per night on weeknights, regardless of weekends, for optimal emotional wellbeing.

## Data Availability

Datasets or materials can be accessed by contacting the corresponding author.

## Footnotes

1 Information on the other measures administered as part of the online survey can be obtained from the first author. The present data have not been included in other studies.
